# Open label, dose escalation trial of creatine monohydrate in pregnancy

**DOI:** 10.1080/15502783.2025.2533652

**Published:** 2025-07-20

**Authors:** Madison Naidu, Deborah L de Guingand, Georgina Hunter, Nhi T Tran, Rod J Snow, Carl M Kirkpatrick, Stacey J Ellery, Kirsten R Palmer

**Affiliations:** a Monash University, Department of Obstetrics and GynaecologyClayton, Victoria, Australia; b The Ritchie Centre, Hudson Institute of Medical Research Clayton, Victoria, Australia; c Deakin University, Institute for Physical Activity & Nutrition, Melbourne, Australia; d Monash University, Monash Institute for Pharmaceutical Sciences, Parkville, Melbourne

**Keywords:** Creatine, pregnancy, pharmacokinetics, dose-response relationship, drug, maternal-fetal exchange, energy metabolism

## Abstract

**Background:**

Preclinical data demonstrates that creatine supplementation during pregnancy supports maternal and fetal energy homeostasis through periods of hypoxia, but its use in human pregnancy remains unexplored. This trial aims to determine optimal dosing regimens of creatine monohydrate (CrM) for pregnant women to achieve maximum steady-state plasma concentrations.

**Methods:**

This was a single-site, open-label, dose-escalation pharmacokinetic trial conducted in two stages. In Stage 1, a single 5g dose of creatine monohydrate (CrM) was administered to healthy non-pregnant women (n=8) and pregnant women in the third trimester (n=7). Plasma creatine concentrations were measured from 0 to 10 hours post-dose to determine peak plasma concentrations. Population pharmacokinetic modelling and Monte Carlo simulations were performed. Stage 2 involved multi-dose administration of 5g CrM every 8 hours for 3 days in pregnant women (n=8). Plasma creatine concentrations were measured pre-dose and 1 hour post the first dose on Day 1, and pre-dose and 1 hour post the last dose on Day 3 to assess drug accumulation. Safety, tolerability, and fetal monitoring were also evaluated.

**Results:**

Pharmacokinetic modelling has been completed for Stage 1, where baseline, Cmax and Tmax creatine concentrations were comparable in pregnant and non-pregnant participants after a 5g CrM dose. Monte Carlo simulations predicted that dosing 5g CrM 3-4 times daily would likely achieve a steady-state plasma concentration of 50mg/L of creatine within 72 hours of commencing CrM administration, while a 10g dose every 12 hours would have a similar effect. Participants reported no major adverse events or side effects.

**Conclusion:**

Pregnancy in the third trimester does not significantly alter the pharmacokinetics of CrM administration compared to non-pregnant females. Dosing regimens consistent with ergogenic trials (5g 3-4 times daily) appear effective for achieving the desired steady-state concentrations in a pregnant population. Including data from Stage 2 will strengthen the model and help validate this conclusion.

